# Diaphragm Thickening Fraction as a Prognostic Imaging Marker for Postoperative Pulmonary Complications in Robot-Assisted Laparoscopic Prostatectomy Requiring the Trendelenburg Position and Pneumoperitoneum

**DOI:** 10.1155/2021/9931690

**Published:** 2021-06-22

**Authors:** Jihion Yu, Yongsoo Lee, Jun-Young Park, Jai-Hyun Hwang, Young-Kug Kim

**Affiliations:** ^1^Department of Anesthesiology and Pain Medicine, Asan Medical Center, University of Ulsan College of Medicine, Seoul, Republic of Korea; ^2^Department of Anesthesiology and Pain Medicine, Uijeongbu Eulji Medical Center, Eulji University School of Medicine, Gyeonggi-do, Republic of Korea

## Abstract

**Background:**

Robot-assisted laparoscopic prostatectomy (RALP) frequently entails postoperative pulmonary complications (PPCs) due to the Trendelenburg position and pneumoperitoneum. Diaphragm thickening fraction (TF) as an imaging marker can offer the advantage of predicting respiratory outcomes. Therefore, we evaluated the effect of diaphragm TF on the occurrence of PPCs in RALP.

**Methods:**

We measured the preoperative thickness of the diaphragm at peak inspiration (*T*_pi_) and end expiration (*T*_ee_) using ultrasonography. Diaphragm TF was calculated as TF = (*T*_pi_–*T*_ee_)/*T*_ee_. A receiver operating characteristic (ROC) curve analysis of TF was performed. After dividing patients into two groups according to the optimal TF cut-off value, we compared the occurrence of PPCs between the groups. The predictivity of diaphragm TF for the occurrence of PPCs was evaluated.

**Results:**

Of 145 patients, 40 patients (27.6%) developed PPCs. Patients with PPCs had a significantly lower TF than those without PPCs (0.31 ± 0.09 vs. 0.39 ± 0.11, *P* < 0.001). In the ROC curve analysis, the optimal TF cut-off value was 0.28. The patients were divided into TF ≥ 0.28 group (*n* = 114) and TF < 0.28 group (*n* = 31). The incidence of PPCs was significantly higher in the TF < 0.28 group than in the TF ≥ 0.28 group (51.6% vs. 21.1%, *P* = 0.001). Diaphragm TF < 0.28 was associated with a higher incidence of PPCs than diaphragm TF ≥ 0.28 (odds ratio = 4.534, 95% confidence interval [1.763–11.658], *P* = 0.002).

**Conclusion:**

Preoperative diaphragm TF < 0.28 was associated with an increased incidence of PPCs, suggesting that diaphragm TF as a prognostic imaging marker provides useful information on PPCs in RALP requiring the Trendelenburg position and pneumoperitoneum. *Trial Registry Number*. This trial is registered with KCT0005028.

## 1. Introduction

Robot-assisted laparoscopic prostatectomy (RALP) has been primarily adopted for prostate cancer due to its several advantages over open prostatectomy, including lower intraoperative blood loss, fewer blood transfusions, fewer anastomotic strictures, and shorter hospital stay [1]. However, RALP requires the steep Trendelenburg position and carbon dioxide pneumoperitoneum to maintain a good surgical condition. These specific surgical conditions reduce the functional residual capacity, vital capacity, and lung compliance [2, 3]. Consequently, in patients undergoing RALP, these conditions can adversely affect the respiratory system and lead to postoperative pulmonary complications (PPCs) [2, 3]. Furthermore, PPCs are associated with increased morbidity and mortality rates, even mild PPCs, such as atelectasis [4–6]. Therefore, meticulous preoperative evaluation and perioperative management are required to reduce PPCs in RALP.

The diaphragm is a principal muscle of respiration, and its function can be evaluated by diaphragm thickening fraction (TF) during respiration at the zone of apposition, which is the area of the diaphragm where it begins to peel away from the lower rib cage [7]. Diaphragm TF, which can be simply measured at bedside by ultrasonography, can precisely reflect the invasive gold standard measure of diaphragm function (i.e., transdiaphragmatic pressure measurement) [8, 9]. In particular, a low diaphragm TF can be a predictor of the failure of mechanical ventilation weaning in the intensive care unit and is associated with PPCs in cardiac surgery [10, 11]. However, little is known about the association between diaphragm TF and PPCs in surgeries requiring the steep Trendelenburg position and carbon dioxide pneumoperitoneum that adversely affect the respiratory system.

In this study, we hypothesized that low diaphragm TF can predict PPCs in RALP, which requires carbon dioxide pneumoperitoneum and the steep Trendelenburg position. To this end, after dividing the patients into two groups according to the optimal TF cut-off value for predicting PPCs, we evaluated the effect of low diaphragm TF as an imaging marker on the occurrence of PPCs in RALP.

## 2. Patients and Methods

### 2.1. Methods

This prospective observational study was conducted at a tertiary referral center. Prior to patient enrollment, the study protocol was registered at the Clinical Research Information Service (KCT 0005028, principal investigator: Young-Kug Kim, registration date: May 18, 2020). All patients provided written informed consent prior to study participation. The institutional review board of Asan Medical Center (Seoul, South Korea) approved the study protocol (approval number: 2020-0761, approval date: May 15, 2020).

### 2.2. Study Population

The study patients were enrolled between May 2020 and September 2020. The inclusion criteria were age 20–79 years, scheduled RALP, American Society of Anesthesiologists (ASA) physical status I–III, and voluntary participation in this study. Patients who underwent pneumonectomy and those who were converted to open prostatectomy were excluded.

### 2.3. Study Protocol

All patients were monitored with pulse oximetry, electrocardiography, end-tidal carbon dioxide concentration, noninvasive blood pressure, and bispectral index (A-1050 Monitor; Aspect Medical Systems, Newton, MA, USA). Anesthesia was induced using thiopental sodium (4–5 mg/kg) and rocuronium (0.5–0.8 mg/kg) and maintained with sevoflurane (1–2 vol%), medical air containing 50% oxygen, and 1.0–4.0 ng/mL remifentanil. The ventilation setting was a tidal volume of 6–8 mL/kg of the ideal body weight and an inspiratory-to-expiratory ratio of 1 : 2. Respiratory rate of 10–16 cycles/min was adjusted to achieve an end-tidal carbon dioxide partial pressure of 35–45 cmH_2_O but not to exceed the maximum peak airway pressure of 30 cmH_2_O. Positive end-expiratory pressure was applied at 6 cmH_2_O with a recruitment maneuver (40 cmH_2_O airway pressure for 30 seconds). The depth of anesthesia was maintained with the bispectral index of 40–60. Fluids and vasopressors, such as ephedrine and phenylephrine, were administered to maintain systolic blood pressure above 80 mmHg. Patients were administered crystalloid fluid at 2–4 mL/kg/h. Train-of-four monitoring was used to measure the degree of neuromuscular blockade. Rocuronium bromide was intermittently administered to maintain train-of-four count ≤ 2 throughout surgery. RALP was performed using the standard techniques of our institution [12, 13]. Pneumoperitoneum was induced with carbon dioxide gas at 15 mmHg abdominal pressure. Patients were positioned in the steep Trendelenburg position (45 degrees) during RALP. After skin closure, 2 mg/kg sugammadex (Bridion®; MSD, Oss, the Netherlands) was used to reverse the neuromuscular blockade if the train-of-four count was ≥ 2 at the end of surgery.

### 2.4. Measurements

**Figure 1:**
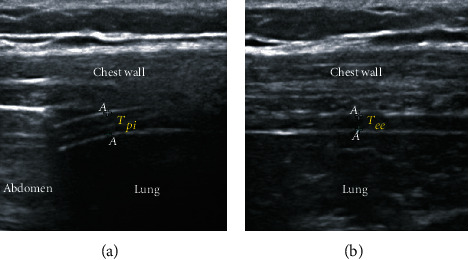
Measurement of diaphragm thickening fraction (TF) by ultrasonography. The diaphragm thicknesses at peak inspiration (*T*_pi_) (a) and end expiration (*T*_ee_) (b) were measured. Diaphragm TF was calculated as follows: TF = (*T*_pi_–*T*_ee_)/*T*_ee_.

Diaphragm TF was measured using a 13-6 MHz linear transducer (Sonosite X-Porte; Fujifilm SonoSite, Bothell, WA, USA) before anesthesia induction by two investigators highly experienced in lung and diaphragm ultrasonography. Patients were placed in a semirecumbent position with the head of the bed tilted downward at 45 degrees. The probe was placed on the ninth or tenth intercostal space in the left and right midaxillary line and perpendicularly angled to the chest wall. A 2D-clip (B-mode) was acquired while the patient performed a maximal inspiration and expiration. The diaphragm thicknesses at peak inspiration (*T*_pi_) and end expiration (*T*_ee_) were measured on the clip. Diaphragm TF was calculated as TF = (*T*_pi_–*T*_ee_)/*T*_ee_ (Figure 1) [8]. We acquired the measurements twice for both left and right sides and used the average of all four values in the analysis. Interobserver variability was calculated by evaluating a random sample of approximately 25% (i.e., 37/145 patients) of diaphragm TF twice by two investigators. Intraobserver variability was calculated by evaluating a random sample of approximately 25% of diaphragm TF twice by one investigator. The interobserver and intraobserver variabilities were determined as the mean absolute difference between the two values divided by their mean and presented as a percentage.

### 2.5. Assessments

Preoperative data included age, body mass index, ASA physical status, hypertension, diabetes mellitus, cerebrovascular disease, coronary artery disease, interstitial lung disease, chronic obstructive pulmonary disease, pulmonary tuberculosis, and preoperative pulmonary function test findings. Intraoperative data included preinduction hemodynamics, such as mean blood pressure, systolic blood pressure, diastolic blood pressure, body temperature, heart rate, and peripheral oxygen saturation, arterial blood gas analyses before induction and at skin closure, anesthesia duration, operation duration, and crystalloid amount. Arterial blood gas analysis included arterial hydrogen ion concentration (pH), arterial oxygen partial pressure (PaO_2_), arterial carbon dioxide partial pressure (PaCO_2_), bicarbonate (HCO_3_^−^), base excess, and arterial oxygen saturation.

Postoperative variables included PPCs and the duration of hospitalization defined as the period from the day of surgery to the day of discharge. PPCs were diagnosed as the occurrence of the following within seven days after RALP [14–20]: atelectasis, pleural effusion, bronchospasm, pneumothorax, respiratory infection, aspiration pneumonitis, and respiratory failure (Table 1).

### 2.6. Statistical Analysis

By referring to our previous study [15], we assumed a normal distribution, correlation of 0.2 with other variables, and 30% occurrence of PPCs in RALP. Accordingly, it was calculated that 145 patients were needed to detect an odds ratio of 2.0 for a low diaphragm TF with 90% power, a two-sided *α* = 0.05, and a 10% dropout rate. Categorical variables were compared using the Fisher's exact test or the chi-squared test as appropriate and are presented as number (percentage). Continuous variables were compared using the Mann–Whitney *U* test or unpaired *t*-test as appropriate and are presented as mean ± standard deviation.

To determine the prognostic ability of preoperative diaphragm TF for the occurrence of PPCs in RALP, the receiver operator characteristic (ROC) curve analysis was performed. The value with the highest specificity and sensitivity was designated as the optimal cut-off value. After dividing into two groups according to the optimal diaphragm TF cut-off value for predicting PPCs (i.e., low TF vs. high TF), we compared the occurrence of PPCs between high and low TFs in RALP. The predictive ability of low diaphragm TF for the occurrence of PPCs was evaluated by a multivariate-adjusted odds ratio. A *P* value of < 0.05 was considered to denote statistical significance. IBM SPSS version 21.0.0 for Windows (IBM Corp., Armonk, NY, USA) and MedCalc version 11.3.3.0 (MedCalc Software bvba, Mariakerke, Belgium) were used for statistical analyses.

## 3. Results

**Figure 2:**
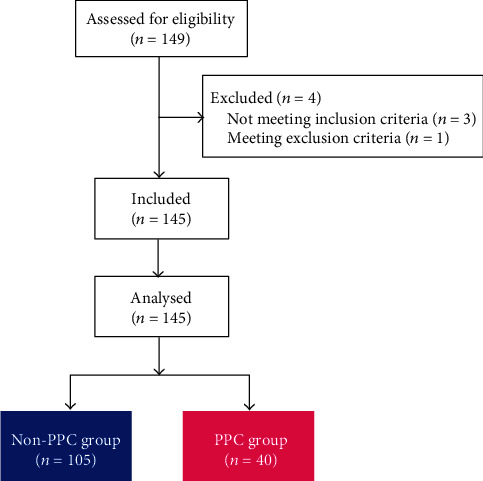
Study flow diagram of patients. PPC: postoperative pulmonary complication.

A total of 149 patients were preoperatively assessed for eligibility, of whom 145 patients were finally included in the analysis after excluding four patients (Figure 2). Forty patients (27.6%) developed PPCs. One patient developed pneumonia, 22 patients developed pleural effusion, and 27 patients developed atelectasis.  Table 2 enlists the preoperative and intraoperative data. The PPC group and the non-PPC group did not show significant differences in preoperative and intraoperative data (Table 2).

**Figure 3:**
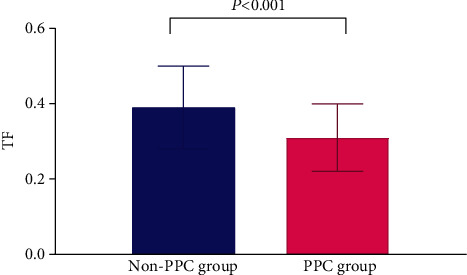
Comparison of the preoperative diaphragm TF between the PPC group and non-PPC group in patients undergoing RALP. Note that diaphragm TF is significantly lower in the PPC group than in the non-PPC group. TF: thickening fraction; PPC: postoperative pulmonary complication; RALP: robot-assisted laparoscopic prostatectomy.

**Figure 4:**
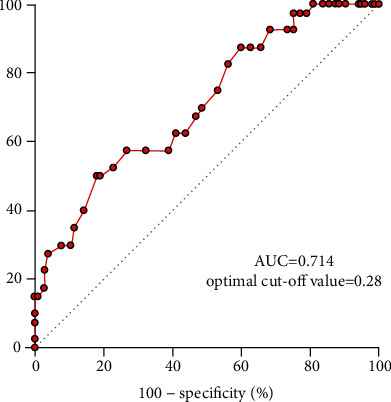
Receiver operating characteristic curve analysis of diaphragm TF for predicting PPCs in patients undergoing RALP. The AUC is 0.714, with an optimal cut-off value of 0.28. TF: thickening fraction; PPCs: postoperative pulmonary complications; RALP: robot-assisted laparoscopic prostatectomy; AUC: area under the curve.

**Figure 5:**
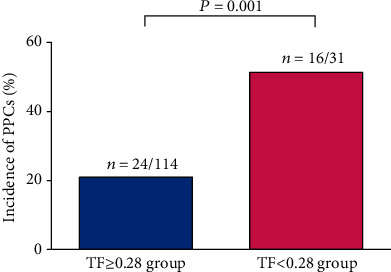
Comparison of the incidence of PPCs between the TF ≥ 0.28 group and TF < 0.28 group. Note that the TF < 0.28 group has a higher incidence of PPCs than TF ≥ 0.28 group. PPCs: postoperative pulmonary complications; TF: thickening fraction.

**Figure 6:**
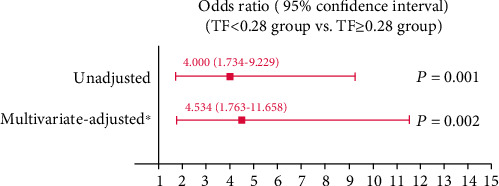
Predictive ability of diaphragm TF ≥ 0.28 for the occurrence of PPCs in patients undergoing RALP. ∗The multivariate-adjusted odds ratio was adjusted using the variables shown in Table 2. TF: thickening fraction; PPCs: postoperative pulmonary complications; RALP: robot-assisted laparoscopic prostatectomy.

Preoperative diaphragm TF was significantly lower in the PPC group than in the non-PPC group (0.31 ± 0.09 vs. 0.39 ± 0.11, *P* < 0.001) (Figure 3). No significant differences were found between TFs in the right and left diaphragm (0.36 ± 0.14 vs. 0.38 ± 0.13, *P* = 0.167). The interobserver and intraobserver variabilities of diaphragm TF measurements were 2.2% and 2.4%, respectively. In ROC curve analysis, the area under the curve of diaphragm TF for predicting PPCs was 0.714 (Figure 4). The optimal diaphragm TF cut-off value for predicting the occurrence of PPCs was 0.28. The patients were then categorized according to the optimal diaphragm TF cut-off value: TF ≥ 0.28 group (*n* = 114) and TF < 0.28 group (*n* = 31).  Table 3 enlists preoperative and intraoperative data of the two groups. There were no significant differences in preoperative and intraoperative data between TF ≥ 0.28 and TF < 0.28 groups (Table 3). The incidence of PPCs was significantly higher in the TF < 0.28 group than in the TF ≥ 0.28 group (51.6% [16/31] vs. 21.1% [24/114], *P* = 0.001; Figure 5). Compared with the TF ≥ 0.28 group, the TF < 0.28 group had higher incidences of PPCs in unadjusted (odds ratio = 4.000, 95% confidence interval [1.734–9.229], *P* = 0.001) and multivariate-adjusted analyses (odds ratio = 4.534, 95% confidence interval [1.763–11.658], *P* = 0.002; Figure 6). The duration of hospitalization was not significantly different between the PPC group and the non-PPC group (7.9 ± 4.4 days vs. 7.3 ± 1.8 days, *P* = 0.304).

## 4. Discussion

In this prospective study, 40/145 patients (27.6%) developed PPCs in RALP. We found that the patients who developed PPCs had a significantly lower preoperative diaphragm TF than those who did not develop PPCs in RALP performed with specific conditions of the Trendelenburg position and pneumoperitoneum. The optimal cut-off value of preoperative diaphragm TF for predicting PPCs in RALP was 0.28. The incidence of PPCs was significantly higher in the TF < 0.28 group than in the TF ≥ 0.28 group, and diaphragm TF < 0.28 was associated with increased incidence of PPCs in RALP. To our knowledge, our study is the first to present the empirical evidence that preoperative low diaphragm TF as a prognostic imaging marker is associated with a high occurrence of PPCs when RALP is performed with specific intraoperative conditions of the Trendelenburg position and pneumoperitoneum, which confer unfavorable effects on the respiratory system.

In this large prospective observational study, the incidence of PPCs in prostate cancer patients who underwent RALP was 27.6%. Previous studies have reported PPC occurrence rates of 2.7%–33.4% in noncardiac surgeries [5, 21, 22]. Our findings may have been influenced by the aforementioned special surgical conditions and patient characteristics. RALP warrants the surgical conditions of carbon dioxide pneumoperitoneum and the steep Trendelenburg position, which adversely affect the respiratory system [15]. The steep Trendelenburg position can reduce lung compliance and lung volume parameters, such as functional residual capacity and vital capacity; furthermore, it can cause edema of the upper airway and elevation in ventilation–perfusion mismatch and peak airway pressure. These conditions may be aggravated further by carbon dioxide pneumoperitoneum, which causes hypercapnia and respiratory acidosis [23]. Moreover, most patients undergoing RALP are elderly and are thus even more vulnerable under the specific surgical conditions due to decreased lung compliance and pulmonary function [24]. Therefore, patients undergoing RALP show relatively a higher incidence of PPCs compared with those undergoing other noncardiac surgeries [15].

We found that low diaphragm TF was associated with high occurrence of PPCs in RALP requiring the steep Trendelenburg position and pneumoperitoneum. Diaphragm TF at the zone of apposition allows for direct visualization of the diaphragm muscle; as such, the diaphragm TF depends on the activity of the diaphragm and reflects the work of breathing of the diaphragm [7]. In addition, it could be well correlated with overall respiratory function and offers valuable information, such as ventilator-induced diaphragmatic dysfunction and the prediction of difficult weaning in ventilated patients [10, 25]. Moreover, unlike other tools for evaluating the diaphragm function, such as diaphragmatic electromyography, transdiaphragmatic pressure measurement, magnetic resonance imaging, phrenic nerve stimulation, and computed tomography, the ultrasonographic assessment of diaphragm function as an imaging marker is a real-time and noninvasive method with high feasibility and reproducibility and can be done at bedside in awake patients [8, 9].

Importantly, the strength of respiratory muscles is associated with lung reexpansion after surgery [11]. It is known that evaluating the diaphragm function reflects the strength of the inspiratory muscle [26]. Therefore, diaphragmatic dysfunction could contribute to the etiology of PPCs after noncardiac surgery. In particular, the function of the diaphragm could be characterized by diaphragm TF [11, 26, 27]. In agreement with our results, in several previous studies, diaphragm TF was measured using ultrasonography for evaluating diaphragm function in different clinical settings, such as cardiac surgery and intensive care unit setting [11, 27]. Vivier et al. reported that ultrasonographic assessment of diaphragm TF could evaluate the diaphragmatic function and respiratory workload in critically ill patients with noninvasive ventilation [27]. In addition, Cavayas et al. reported that low diaphragm TF was a risk factor for PPCs after cardiac surgery [11, 27]. Therefore, to minimize the risk of PPCs in patients undergoing RALP under carbon dioxide pneumoperitoneum and the steep Trendelenburg position, special attention should be given to patients with low preoperative diaphragm TF.

In our analysis, the optimal cut-off value of the preoperative diaphragm TF in terms of the prediction of PPCs in RALP was 0.28. In line with the results of our study, Dubé et al. demonstrated that a diaphragm TF of less than 0.29 was significantly associated with poor diaphragm strength and could predict prolonged mechanical ventilation and higher intensive care unit and hospital deaths [28]. However, the optimal cut-off value of diaphragm TF for predicting PPCs in RALP is yet to be evaluated, and our current study is the first to report that patients with TF < 0.28 are at a higher risk of PPCs in RALP performed under the Trendelenburg position and pneumoperitoneum.

Respiratory muscles include expiratory muscles (e.g., diaphragm and internal intercostal muscles) and inspiratory muscles (e.g., diaphragm and external intercostal muscles) [8]. The reduced respiratory muscle capacity contributes to pulmonary complications in critically ill patients [29]. In particular, the diaphragm is the main respiratory muscle that has a key role in pulmonary complications [8]. Therefore, measuring diaphragm TF using ultrasonography can properly reflect the active contraction of respiratory muscle bundles and help assess the diaphragm activity and function [30, 31]. Furthermore, ultrasonography could be simply and noninvasively performed at bedside. Taken together, our study has its strengths because we evaluated the ultrasonographic diaphragm TF for predicting PPCs in a large number of patients undergoing RALP under the Trendelenburg position and pneumoperitoneum.

Our study is limited in that it was conducted in a single center and may have limited generalizability; therefore, multicenter studies are needed for further validation of the results. In addition, our study did not specifically evaluate the relationship between PPCs and diaphragm TF in patients undergoing other types of surgeries, which should be investigated in further targeted studies.

## 5. Conclusions

PPCs occurred in 27.6% of patients who underwent RALP requiring carbon dioxide pneumoperitoneum and the steep Trendelenburg position. Diaphragm TF < 0.28 was associated with a higher likelihood of PPCs in RALP. These results suggest that the evaluation of preoperative diaphragm TF as an imaging marker should be recommended in prostate cancer patients undergoing RALP for predicting the risk of PPCs.

## Figures and Tables

**Table 1:** Diagnostic criteria for postoperative pulmonary complications in robot-assisted laparoscopic prostatectomy.

Complication	Definition
Atelectasis	Atelectasis was defined as lung opacification with a shift of the hilum, hemidiaphragm, or mediastinum toward the affected side and compensatory overinflation in the adjacent nonatelectatic lung.
Pleural effusion	Pleural effusion was defined as chest X-ray showing loss of the sharp silhouette of the ipsilateral hemidiaphragm in the upright position, evidence of displacement of adjacent anatomical structures, blunting of the costophrenic angle, or a hazy opacity in one hemithorax with preserved vascular shadows.
Bronchospasm	Bronchospasm was defined as newly developed expiratory wheezing that needed treatment with bronchodilators.
Pneumothorax	Pneumothorax was defined as air in the pleural space without vascular bed surrounding the visceral pleura.
Respiratory infection	Respiratory infection was diagnosed as the need of treatment with antibiotics for suspected respiratory infection and as the occurrence of one or more of the following symptoms: new or changed sputum, fever, new or changed lung opacities, or leukocyte count more than 12,000/mm^3^.
Aspiration pneumonitis	Aspiration pneumonitis was defined as an acute lung injury due to aspiration of gastric contents.
Respiratory failure	Respiratory failure was defined as a partial arterial oxygen pressure/fractional inspired oxygen concentration < 300 mmHg, partial arterial oxygen pressure < 60 mmHg in room air, or arterial oxygen saturation measured with pulse oximeter < 90% and requiring oxygen therapy.

Atelectasis, pleural effusion, and pneumothorax were diagnosed with radiologist's description of chest X-rays.

**Table 2:** Preoperative and intraoperative data.

Variables	All patients (*n* = 145)	Non-PPC group (*n* = 105)	PPC group (*n* = 40)	*P* value^∗^

Age (years)	67.2 ± 6.3	67.0 ± 6.7	67.9 ± 5.2	0.418
Body mass index (kg/m^2^)	24.8 ± 2.6	24.8 ± 2.7	24.9 ± 2.4	0.912
ASA physical status				0.637
2	119 (82.1)	85 (81.0)	34 (85.0)	
3	26 (17.9)	20 (19.0)	6 (15.0)	
Hypertension	61 (42.1)	46 (43.8)	15 (37.5)	0.492
Diabetes mellitus	20 (13.8)	15 (14.3)	5 (12.5)	0.800
Cerebrovascular disease	10 (6.9)	9 (8.6)	1 (2.5)	0.285
Coronary artery disease	14 (9.7)	9 (8.6)	5 (12.5)	0.532
COPD	19 (13.1)	14 (13.3)	5 (12.5)	>0.999
Interstitial lung disease	0 (0)	0 (0)	0 (0)	>0.999
Pulmonary tuberculosis	0 (0)	0 (0)	0 (0)	>0.999
Pulmonary function test				
FVC (L)	3.8 ± 0.6	3.8 ± 0.6	3.6 ± 0.6	0.104
FEV_1_ (L)	2.8 ± 0.5	2.8 ± 0.5	2.6 ± 0.5	0.051
FEV_1_/FVC ratio (%)	73.2 ± 11.2	73.6 ± 11.7	72.2 ± 10.1	0.477
Pre-induction hemodynamics				
Mean blood pressure (mmHg)	90 ± 11	90 ± 10	90 ± 12	0.743
Systolic blood pressure (mmHg)	137 ± 18	136 ± 18	138 ± 19	0.437
Diastolic blood pressure (mmHg)	75 ± 11	75 ± 10	75 ± 13	0.728
Body temperature (°C)	36.6 ± 0.3	36.6 ± 0.3	36.6 ± 0.3	0.850
Heart rate (beats/min)	71 ± 13	70 ± 13	71 ±12	0.642
SpO_2_ (%)	97.9 ± 1.8	98.0 ± 1.8	97.7 ± 1.8	0.343
Arterial blood gas analysis after induction				
pH	7.46 ± 0.03	7.46 ± 0.03	7.46 ± 0.03	0.722
PaO_2_ (mmHg)	288.4 ± 72.8	287.5 ± 70.9	290.6 ±78.6	0.822
PaCO_2_ (mmHg)	39.1 ± 4.8	39.2 ± 5.0	38.9 ± 4.1	0.713
HCO_3_^−^ (mmol/L)	28.1 ± 2.3	28.2 ± 2.3	27.7 ± 2.3	0.248
Base excess (mmol/L)	4.0 ± 2.1	4.1 ± 2.1	3.7 ± 2.1	0.236
SaO_2_ (%)	99.9 ± 0.5	99.9 ± 0.4	99.9 ± 0.6	0.566
Arterial blood gas analysis at skin closure				
pH	7.42 ± 0.03	7.42 ± 0.03	7.41 ± 0.04	0.055
PaO_2_ (mmHg)	189.0 ± 45.0	191.8 ± 43.4	181.7 ± 48.8	0.230
PaCO_2_ (mmHg)	40.5 ± 3.3	40.2 ± 3.2	41.3 ± 3.3	0.071
HCO_3_^−^ (mmol/L)	26.1 ± 1.9	26.1 ± 1.9	26.2 ± 2.2	0.934
Base excess (mmol/L)	1.5 ± 2.0	1.6 ± 1.9	1.3 ± 2.4	0.408
SaO_2_ (%)	99.6 ± 0.8	99.7 ± 0.6	99.4 ± 1.0	0.051
Anesthesia duration (min)	160.9 ± 25.8	160.9 ± 24.6	160.9 ± 28.9	0.988
Operation duration (min)	120.8 ± 25.6	120.8 ± 24.5	121.0 ± 28.8	0.963
Crystalloid amount (mL)	838.7 ± 540.5	848.2 ± 596.9	813.8 ± 357.3	0.733

Continuous variables are presented as mean ± standard deviation, and categorical variables are presented as number (percentage). ∗ For comparisons between the PPC and non-PPC groups. ASA, American Society of Anesthesiologists; COPD, chronic obstructive pulmonary disease; FEV_1_, forced expiratory volume in the first second; FVC, forced vital capacity; SpO_2_, peripheral oxygen saturation; pH, hydrogen ion concentration; PaCO_2_, arterial carbon dioxide partial pressure; PaO_2_, arterial oxygen partial pressure; HCO_3_^−^, bicarbonate; SaO_2_, arterial oxygen saturation.

**Table 3:** Preoperative and intraoperative data of the two groups categorized according to the optimal diaphragm TF cut-off value.

Variables	TF ≥ 0.28 group (*n* = 114)	TF < 0.28 group (*n* = 31)	*P* value
Age (years)	67.5 ± 6.1	66.3 ± 7.0	0.349
Body mass index (kg/m^2^)	24.8 ± 2.6	24.9 ± 2.5	0.926
ASA physical status			0.598
2	92 (80.7)	27 (87.1)	
3	22 (19.3)	4 (12.9)	
Hypertension	42 (43.0)	12 (38.7)	0.838
Diabetes mellitus	16 (14.0)	4 (12.9)	>0.999
Cerebrovascular disease	8 (7.0)	2 (6.5)	>0.999
Coronary artery disease	12 (10.5)	2 (6.5)	0.388
COPD	14 (12.3)	5 (16.1)	0.765
Interstitial lung disease	0 (0)	0 (0)	>0.999
Pulmonary tuberculosis	0 (0)	0 (0)	>0.999
Pulmonary function test			
FVC (L)	3.8 ± 0.6	3.8 ± 0.7	0.731
FEV_1_ (L)	2.8 ± 0.5	2.8 ± 0.7	0.549
FEV_1_/FVC ratio (%)	73.1 ± 11.2	73.7 ± 11.5	0.796
Preinduction hemodynamics			
Mean blood pressure (mmHg)	90.5 ± 10.3	87.6 ± 12.1	0.173
Systolic blood pressure (mmHg)	137.0 ± 18.2	134.7 ± 17.6	0.538
Diastolic blood pressure (mmHg)	76.0 ± 10.4	72.0 ± 12.2	0.075
Body temperature (°C)	36.6 ± 0.3	36.6 ± 0.3	0.943
Heart rate (beats/min)	70.6 ± 12.9	70.4 ± 12.1	0.931
SpO_2_ (%)	98.0 ± 1.8	97.5 ± 1.6	0.140
Arterial blood gas analysis after induction			
pH	7.5 ± 0.03	7.5 ± 0.03	0.472
PaO_2_ (mmHg)	283.9 ± 70.6	304.7 ± 79.4	0.193
PaCO_2_ (mmHg)	38.9 ± 4.9	40.0 ± 4.5	0.235
HCO_3_^−^ (mmol/L)	28.0 ± 2.2	28.4 ± 2.7	0.442
Base excess (mmol/L)	4.0 ± 2.0	4.2 ± 2.4	0.648
SaO_2_ (%)	99.9 ± 0.4	99.9 ± 0.7	0.709
Arterial blood gas analysis at skin closure			
pH	7.4 ± 0.04	7.4 ± 0.05	0.460
PaO_2_ (mmHg)	188.9 ± 43.6	189.6 ± 50.5	0.939
PaCO_2_ (mmHg)	40.2 ± 3.3	41.3 ± 3.0	0.093
HCO_3_^−^ (mmol/L)	26.2 ± 1.9	26.0 ± 2.2	0.625
Base excess (mmol/L)	1.6 ± 1.9	1.2 ± 2.4	0.412
SaO_2_ (%)	99.6 ± 0.7	99.4 ± 1.0	0.070
Anesthesia duration (min)	159.3 ± 24.8	166.7 ± 28.6	0.200
Operation duration (min)	119.4 ± 24.9	126.2 ± 28.0	0.192
Crystalloid amount (mL)	845.7 ± 577.3	812.9 ± 382.1	0.766

Continuous variables are presented as mean ± standard deviation, and categorical variables are presented as number (percentage). TF: thickening fraction; ASA: American Society of Anesthesiologists; COPD: chronic obstructive pulmonary disease; FEV_1_: forced expiratory volume in the first second; FVC: forced vital capacity; SpO_2_: peripheral oxygen saturation; pH: hydrogen ion concentration; PaCO_2_: arterial carbon dioxide partial pressure; PaO_2_: arterial oxygen partial pressure; HCO_3_^−^: bicarbonate; SaO_2_: arterial oxygen saturation.

## Data Availability

The data used in the present study are available from the corresponding author upon reasonable request.
